# Self-assembly of copper and cobalt complexes with hierarchical size and catalytic properties for hydroxylation of phenol

**DOI:** 10.1186/1556-276X-6-484

**Published:** 2011-08-08

**Authors:** Huaze Dong, Wenbo Tao, Jianhong Bi, Victoria Milway, Zhiqiang Xu, Shengyi Zhang, Xiangchun Meng, Wentao Bi, Jian Li, Meng Li

**Affiliations:** 1 Department of Chemistry and Chemical Engineering, Hefei Normal University, Hefei 230061, People's Republic of China; 2 School of Chemistry and Chemical Engineering, Anhui University, Hefei 230039, People's Republic of China; 3 School of Chemistry, University of Glasgow, University Ave., Glasgow G12 8QQ, UK; 4 SKC PowerTech, Inc., 850 Clark Drive, Mt. Olive, NJ 07828, USA

**Keywords:** self-assembly, coordination polymer, catalysis, hydroxylation

## Abstract

A feasible and effective self-assembly method to synthesize different scale coordination polymers in highly dilute solution (from nanocrystals to microcrystals and to bulk crystals) without any blocking agent has been described. The growth of crystalline particles was controlled by removing the particles at different reaction times to interrupt the growth at the desired size. The nano and microscale particles show better catalytic conversions and selectivities in the hydroxylation of phenols than the bulk crystals.

## Introduction

Within the large family of materials science, nano- and microscale materials are of particular interest for their potential applications in many areas [1-10]. In many cases, these materials exhibit an increase of macroscopic properties due to the microscopic size effect and to the surface effect [11-13]. However, to date, the vast majority of work on nano- or micro-materials has concentrated on purely inorganic compounds or clusters [14-16]. Recently, along with the development of metal-organic framework materials [17,18], a new class of functionalized organic-inorganic hydrid nanomaterial, commonly called microscale coordination polymers (MCPs) [19-21], has received great attention. These materials exhibit a higher level of structural tailorability, with size- and morphology-dependent properties. These structures can also exhibit microporosity, tunable fluorescence, magnetic susceptibility, and unusual catalytic activity and selectivity. Thus far, a variety of methods now exists for making numerous compositions with modest control over particle size and shape as well as properties. There are two very different strategies to synthesize catalytically active MCPs. In the first approach, the metal centers have unsaturated coordination environments which are utilized as catalytically active sites. In the other approach, catalytic sites are incorporated directly into the bridging ligands used to construct MCPs [22,23]. For synthesis of nanomaterials with a specific shape and function, it often uses a soft or hard template. However in the absence of a template, solution-based methods for producing low-dimensional structures require precise tuning of nucleation and growth steps to achieve crystallographic control. These processes are governed by thermodynamic (e.g., temperature, reduction potential) and kinetic (e.g., reactant concentration, diffusion, solubility, reaction rate) parameters [6]. Recently some researchers have reported methods to control the particle shape by the addition of a blocking agent or no blocking agent [8-10]. However, there is still no facile way to microfabricate hierarchical MCPs and there are few examples of such MCPs with catalytic properties. Therefore it is attractive to establish a synthetic strategy for the preparation of hierarchical MCPs with catalytic activity. To this end, the possibility of producing MCPs from the growing, catalytically active CP is considered. The bulk material always has a growth process and the size of the particle could be controlled by interrupting the growth at different stages in highly dilute solution, which also is a common method to synthesize macrocyclic compounds [24-26].

**Figure 1:**
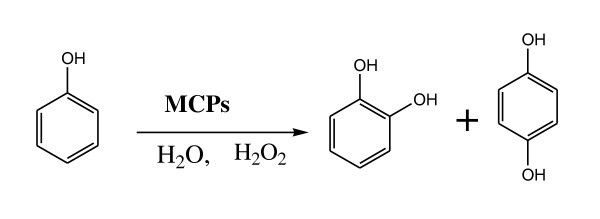
**Catalytic phenol hydroxylation by H2O_2_**.

Hydroxylation of phenols with hydrogen peroxide is a widely used green method of preparing biphenols and is an industrially important reaction for the production of phenol derivatives, which have several large-scale industrial applications in the chemical, pharmaceutical and food industries [27]. Transition metal-based complexes and oxides are well-known catalysts in this reaction [28-34]. Herein, we present the size controllable synthesis of two series of hierarchical MCPs using a simple method under mild conditions. The catalytic activities of the crystalline MCPs were investigated in the hydroxylation of phenol (Figure 1). The as-synthesized MCPs {[M(phen)(C_2_O_4_)(H_2_O)] H_2_O, M = Cu(II) or Co(II), phen = 1,10-phenanthroline} exhibit better catalytic activities than the CP in the hydroxylation of phenols with H_2_O_2 _in aqueous solution and mild conditions, and a high conversion (73.08%, 50°C, 5 h) and high selectivity for hydroquinone with a maximum hydroquinone (HQ)/catechol (CAT) ratio of 3.83.

## Experimental

### Materials and physical measurements

All commercially available chemicals were of reagent grade and used as received without further purification. Analyses for carbon, hydrogen and nitrogen were performed on a Perkin-Elmer 1400C analyzer (PerkinElmer, Waltham, MA, USA). Crystallographic X-ray diffraction data was collected on a Siemens SMART CCD area-detector diffractometer (Siemens, Munich, Germany) equipped with graphite-monochromatic MoKα radiation (λ = 0.71073 Å). Lorentz polarization and absorption corrections were applied. The structures were solved by direct methods and refined with the full-matrix least-squares technique using SHELXTL version 5.1 [35]. Anisotropic thermal parameters were assigned to all non-hydrogen atoms. Organic hydrogen atoms were generated geometrically (C-H 0.96 Å) and refined with isotropic temperature factors. Hydrogen atoms on oxygen atoms were located from difference maps and refined isotropically with geometric AFIX restraints of 0.85-0.95 Å. Powder X-ray diffraction (PXRD) was measured on a X' Pert Pro MPD (Philips Corporation, Holland, Netherlands). Infrared spectrum was recorded on a Nexus-870 spectrometer in the range of 4,000-400 cm^-1 ^using the KBr disk method. Thermogravimetric analysis was performed on a Pyris 1 TGA in the range of 30-700°C with a heating rate of 10°C/min. Scanning electron microscopy (SEM) images were collected on a JEOL-2010 Electron Microscopy (JEOL, Tokyo, Japan). Qualitative and quantitative analysis of hydroxylation products were carried out on an Agilent 1200 liquid chromatograph (Agilent Technologies, Santa Clara, CA, USA). Optical images were collected on a BK51-DP70 All-powerful Microscope. Chromatography column: ZORBAX Eclipse XDB-C18 4.6 × 150 mm, mobile phase: 1% HAc-MeOH (1:1, *v*/*v*); mobile phase velocity, 0.8 ml/min; column temperature, 30°C; sample volume, 20 μl; wavelength of ultraviolet detector, 277 nm.

### Synthesis of two series of hierarchical MCPs materials

Two series of hierarchical MCPs {[M(phen)(C_2_O_4_)(H_2_O)]·H_2_O, phen = 1,10-phenanthroline, M = Cu(II)(MCPs-1) and M = Co(II)(MCPs-2)} were made using the following general procedure: a mixed solution (300 mL, CH_3_CH_2_OH/H_2_O = 1:1, *v*/*v*) of 1,10-phenanthroline (10 mmol) was added to an aqueous solution (250 mL) of CuSO_4 _(10 mmol) or CoSO_4 _(10 mmol) over a period of 30 min, with stirring at 1,000 rpm. After 30 min, sodium oxalate (10 mmol) in 1,000 mL water was added with stirring at 1,400 rpm, and stopped at different times to produce hierarchical materials(20 minutes for nanoscale, 2 h for microscale, more than 6 h for macroscale as well as 7 days for the single crystals). The product was separated by a high speed centrifuge at 16,500 rpm at these times to obtain hierarchical MCPs. Yields for MCPs-1: nano particle, 23%; micro particle, 58%; macro particle, 76%. Analysis calculated for C_14_H_12_CuN_2_O_6_: C, 45.72%; H, 3.29%; N, 7.62%. Found: C, 45.68%; H, 3.34%; N, 7.59%.

The yields for MCPs-2: nano particle, 20%; micro particle, 43%; macro particle, 64%. Analysis calculated for C_14_H_12_CoN_2_O6: C, 46.30%; H, 3.33%; N, 7.71%. Found: C, 46.29%; H, 3.40%; N, 7.68%.

### Catalysis experiments

The as-synthesized particles hardly dissolve in the reaction solution. The catalytic activities for the hydroxylation of phenols were measured in a 100 ml glass reaction flask fitted with a water-cooled condenser. After addition of the reagents, the mixture was heated to 50°C with stirring. Hydrogen peroxide (1.0 ml, 30%, w/v) was added dropwise (over a period of about 30 min) to the magnetically stirred solution at the desired conditions. The course of the reaction and the products were monitored by periodically withdrawing a small sample (20 μl) of the reaction mixture, which was analyzed by liquid chromatography.

## Results and discussion

### Synthesis of MCPs-1 and 2

**Figure 2:**
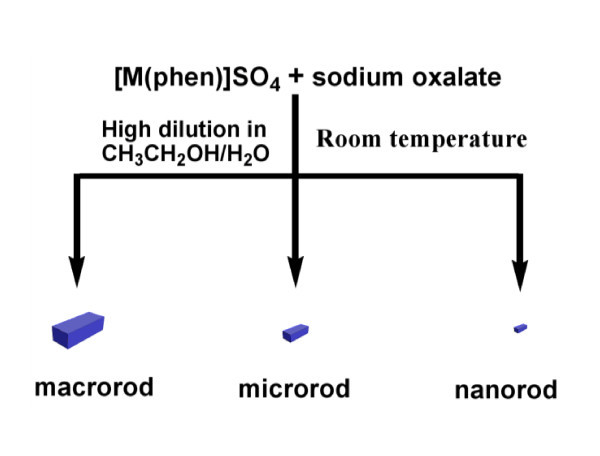
**Growth-phase control of rods using different crystallization times**.

CuSO_4 _or CoSO_4_, 1,10-phenanthroline and sodium oxalate were chosen to synthesize new MCPs. The MCPs were prepared by the stoichiometric reaction of CuSO_4_/CoSO_4 _with 1,10-phenanthroline in mixed solution (CH_3_CH_2_OH:H_2_O = 1:1), followed by reaction with a highly dilute solution of sodium oxalate for different reaction times to achieve the series of products named MCPs (Figure 2).

### Structural characterization of copper and cobalt compounds

**Figure 3:**
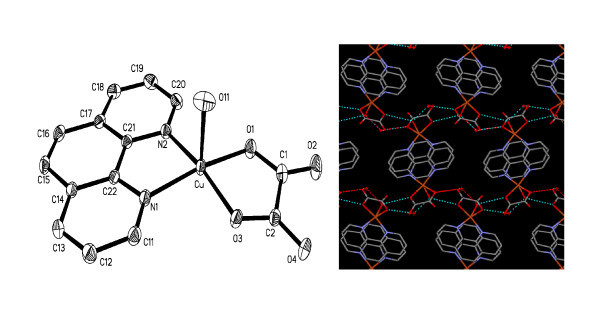
**Mononuclear unit for complex 1 and hydrogen-bonded framework**. (Left) ORTEP view with atom labeling of the mononuclear unit for complex 1 (displacement ellipsoids drawn at 30% probability, H atoms and one lattice water molecule are omitted for clarity,). (Right) Schematic representation of hydrogen-bonded framework of the CPs viewed along *a *axis.

The crystal structure of MCPs-1 with the CCDC number 667029 demonstrates that the copper complex has a mononuclear motif, where each Cu (II) ion is bound by two O atoms from one oxalate anion, one water molecule and two N atoms from one 1,10-phenanthroline, in a slightly distorted square pyramidal coordination geometry (Figure 3, and Additional file 1). It is worth noting that hydrogen bonds and pi-pi stacking interactions play key roles in the fast formation of the hierarchical MCPs-1.

**Figure 4:**
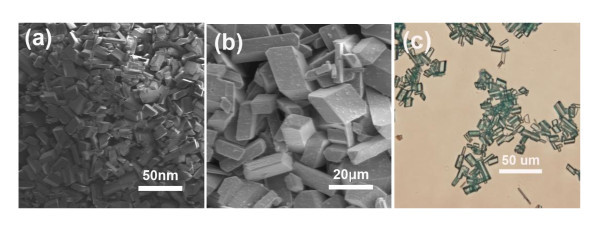
**Images of MCPs-1**. (**a**, **b**) Representative SEM images of the hierarchical MCPs-1: (a) nanoscale CPs and (b) microscale CPs. (**c**) optical image of macroscale CPs.

The experimental conditions were modulated to arrest the polymerization process at early stages to generate nano and microscale MCPs particles [8,34]. The MCPs' size increased from nanorods to microrods and ultimately became macrorods (shown in Figure 4a, b, c) with the increase in crystallization time. When the reaction time reached 20 min, the mixed solution became a little turbid, due to the nano-sized crystalline rods formed. Centrifugal separation of this solution gave blue nano-sized particles and the products have basically the same cuboid morphology (Figure 4a). After about 2 h, microscale crystalline rods were obtained by centrifugal separation of another sample solution (Figure 4b) which has the same morphology as the nanoscale rods. Finally, the third sample underwent 6-h crystallization time, the rods became macroscale (Figure 4c) and single crystals suitable for single crystal X-ray diffraction study were obtained after 1 week at room temperature. The field-emission scanning electron microscopy and optical microscopy images show the growth of crystalline nanorods to microrods and to macrorods. All of the as-synthesized MCPs were insoluble in H_2_O, acetonitrile, methanol, and ethanol.

**Figure 5:**
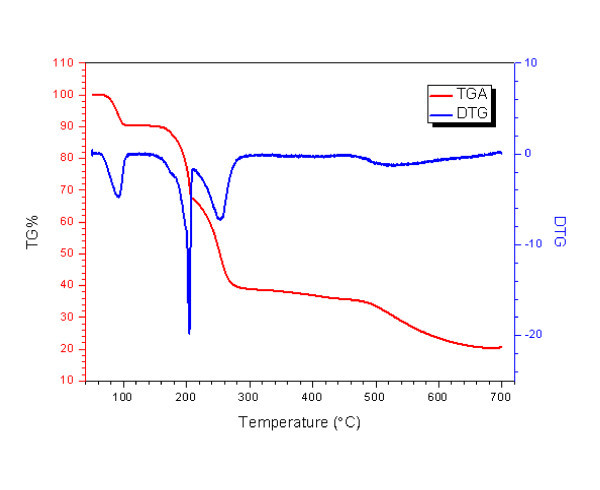
**TGA and DTG curves of the as-synthesized MCPs-1**.

The relative thermal stabilities of the as-synthesized crystalline MCPs-1 were studied using thermogravimetric analysis. Thermogravimetric analysis of products showed the loss of one lattice water and one coordinated water per formula unit in the temperature range 80-100°C, and the loss of the C_2_O_4_^2- ^between 175°C and 205°C. The curve between 205°C and 260°C corresponds to the volatilized phen molecule (Figure 5). It may be concluded that the as-synthesized MCPs-1 is stable between room temperature and 80°C.

**Figure 6:**
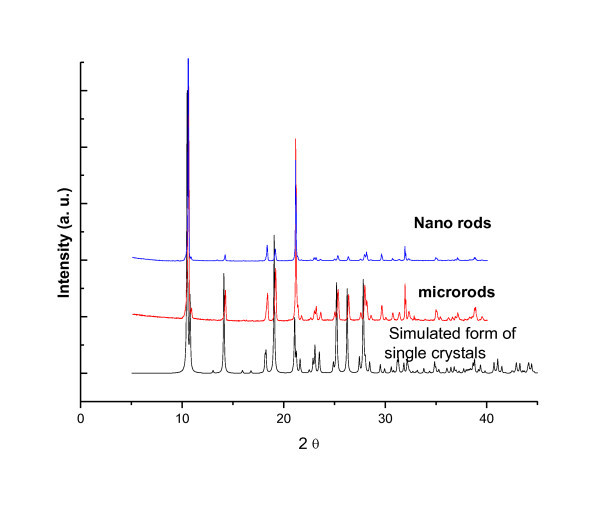
**Comparison of the experimental powder diffraction patterns of MCPs-1**.

Powder X-ray diffraction (PXRD) was also used to investigate the molecular connectivity of MCPs-1. Experimental PXRD patterns of polymorphic particles are presented in Figure 6. All the sharp peaks of the different scale particles mean that they all are crystals. The powder patterns of different scale particles are well coincident with each other and it means that different forms of MCPs-1 have the same structure.

**Figure 7:**
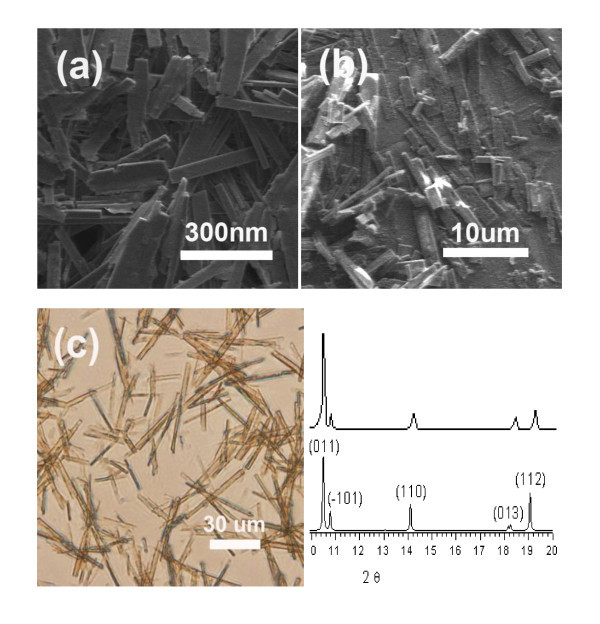
**Images of MCPs-2**. (**a**, **b**) Representative SEM images of the hierarchical MCPs-2: (a) nanoscale CPs and (b) microscale CPs. (**c**) optical image of macroscale CPs, and (**d**) powder XRD patterns: (bottom) simulated XRD pattern, (top) experimental data of XRD pattern.

**Figure 8:**
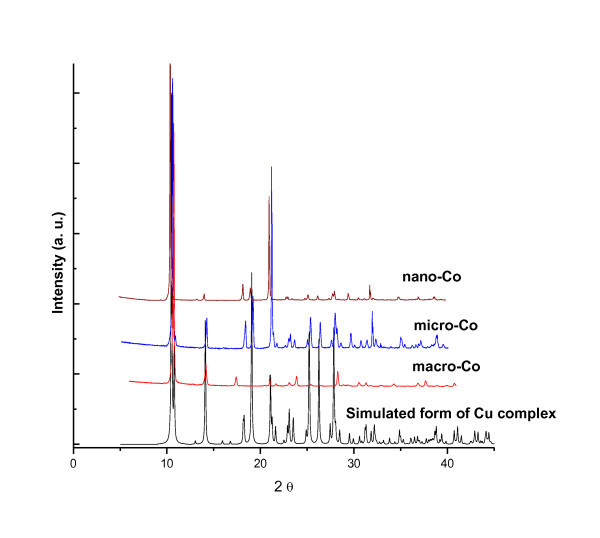
**X-ray diffraction patterns of MCPs-2**.

MCPs-2 was prepared with hierarchical size using a similar method to above MCPs-1 (Figure 7a, b, c). The morphologies of MCPs-2 were regular rectangular plates with well-defined edges. Though single crystals suitable for X-ray crystallography were not obtained, the products studied by powder XRD (Figure 8) have the same chemical connectivity as MCPs-1 in terms of the polymer backbone but a different metal center, as the powder patterns of MCPs-2 are well coincident with the simulated X-ray diffraction pattern calculated from the single crystal data of MCPs-1.

### Catalytic actions of MCPs-1 and 2

To determine suitable reaction conditions for maximum conversion, studies of various reaction parameters were performed (Additional file 2) and the optimum reaction parameters were found to be: the mixture of reagents Phenol (0.2 g) and MCPs catalyst (10 mg) was heated to 50°C with stirring in 40 ml distilled water. Hydrogen peroxide (1.0 ml; 30%, *w*/*v*) was added dropwise to the magnetically stirred solution. Although many kinds of catalyst, such as metal oxides, metal complexes, zeolites, and zeolite-encapsulated metal complexes, have been developed for phenol hydroxylation (and especially for oxidation of phenols), the HQ/CAT ratio generally is much less than that of the MCPs reported here [35-37]. The Italian company Enichem (Rome, Italy) has successfully developed a titanosilicate molecular sieve catalyst (TS-1) with MFI structure for the reaction. The observed conversion of phenol is up to 25%, of which the selectivity of diphenols is about 94% (HQ/CAT = 1:1) [38]. Compared to this TS-1, the MCPs-1 display mild synthetic conditions and exhibit a higher level of structural tailorability and therefore tunable properties via modification of their components (metal ions or ligand). All the as-synthesized MCPs-1 acted as heterogeneous catalysts and consistently showed remarkably high conversion (73% for nanorods at 5 h, 45.79% for microrods, and 36.17% for large crystals) which increased dramatically with a decrease in the size of MCPs-1 (Table 1), and very high selectivity (HQ/CAT = 3.83 for nanorods at 3 h). Furthermore, when the reaction time reached 6 h, the solvent color became a little yellow due to the further oxidation of HQ to cyclohexa-2,5-diene-1,4-dione [39,40]. In a general way, product distribution analysis shows that CAT and HQ are the major reaction products. The series of MCPs-2 exhibits a similar trend to MCPS-1 in activities and product selectivities for the reaction of phenol with H_2_O_2 _(Shown in Table 2). From the two tables, we can see that as the diameter of the heterogeneous catalyst, i.e., MCPs-1 and MCPs-2, was decreased, higher conversions were obtained. The differences in catalytic activity of different-sized particles in the conversion of phenol indicate a significant surface effect of the nano or micro particles, which plays an important role in the conversion of phenol. It was also found that the conversion is much higher for MCPs-1 catalysts than for MCPs-2 catalysts. This implies that MCPs-1 catalysts containing Cu(II) ions are more active than MCPs-2 catalysts containing Co(II) ions. This observation is quite similar to those reported for other complexes [26-32].

**Table 1:** Yields and selectivities of biphenols in the presence of MCPs-1

Catalyst	Conversion (wt.%)		HQ/CAT ratio	
	50°C, 3 h	50°C, 5 h	50°C, 3 h	50°C, 5 h
Nanorods	62.92	73.08	3.83	3.81
Microrods	36.51	45.79	3.79	3.76
Macrorods	28.75	36.17	3.72	3.72

HQ, hydroquinone; CAT, catechol.

**Table 2:** Yields and selectivities of biphenols in the presence of MCPs-2

Catalyst	Conversion (wt.%)		HQ/CAT ratio	
	50°C, 3 h	50°C, 5 h	50°C, 3 h	50°C, 5 h
Nanorods	18.53	23.75	3.56	3.51
Microrods	15.18	19.63	3.55	3.51
Macrorods	9.82	11.97	3.56	3.50

HQ, hydroquinone; CAT, catechol.

**Figure 9:**
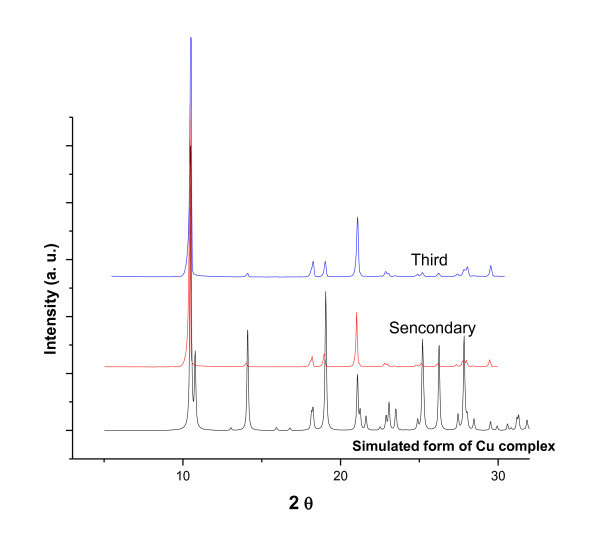
**Comparison of the experimental powder diffraction patterns of the recycled MCPs-1**.

The recyclability of the MCPs-1 has been tested for a typical run, by filtering the reaction mixture after 5 h. The used catalyst was activated by stirring the catalyst with double distilled water for 1 h followed by filtration. This process was repeated twice and the catalyst was dried at 60°C under nitrogen for 1 h. It was reused for a run under similar conditions. It showed similar catalytic activity for the first cycle and there was a very minor loss for the second cycle (Table 3). Experimental PXRD patterns of the used catalyst and fresh catalyst were also nearly the same (Shown in Figure 9). All these studies suggest that the catalyst is sufficiently stable and recyclable. The filtrate collected after separating the used catalyst was placed in the reaction flask and the reaction was continued for another 5 h after adding fresh H_2_O_2_. No significant change was observed in the percentage conversion of phenol.

**Table 3:** The recyclability of the MCPs-1

Catalyst	First time			Second time			Third time		
	Conversion (wt.%) 5 h	HQ/CAT ratio	Rate of recovery, %	conversion (wt.%) 5 h	HQ/CAT ratio	Rate of recovery, %	conversion (wt.%) 5 h	HQ/CAT ratio	Rate of recovery, %
Nanorods	73.08	3.81	75.3	54.75	3.79	63.5	47.53	3.80	72.5
Microrods	45.79	3.76	86.0	42.46	3.78	85.4	41.37	3.74	84.2
Macrorods	36.17	3.72	96.2	36.14	3.51	95.8	35.89	3.50	93.7

HQ, hydroquinone; CAT, catechol.

## Conclusion

In summary, two series of nanocrystals, microcrystals and bulk crystals of MCPs-1 and MCPs-2 have been fabricated by a straightforward and effective self-assembly method in highly dilute solution under mild conditions. In the hydroxylation of phenols, compared with other catalysts, the as-synthesized MCPs displayed a good phenol conversion ratio (73.08%) and high selectivity for the hydroquinone (HQ/CAT ratio of 3.83). The catalyst is stable and recyclable. It can be effectively used as a heterogeneous catalyst.

It was also found that the nature of the central metal ion in the complexes and the surface effect of nano or micro particles have marked effects on phenol conversion according to some reports. The size control method presented herein provides a simple approach for preparation of MCPs with better catalytic properties. It also broadens the range of applications of MCPs materials (Figure 8; Additional files 1 and 2).

## Competing interests

The authors declare that they have no competing interests.

## Authors' contributions

HD, WT and JB carried out the molecular genetic studies, participated in the sequence alignment and drafted the manuscript. SZ and XM, participated in the sequence alignment and performed the structural analysis. WB, JL and ML carried out the catalytic actions of MCPs-1 and 2. VM and ZX participated in the argument on this manuscript and the manuscript was touched up by them. All authors read and approved the final manuscript.

## Supplementary Material

Additional file 1 **Crystal Data of MCPs-1**. crystal data.rar, 51 K. Click here for file

Additional file 2 **Studies of Various Reaction Parameters**. supp1.doc, 56 K. Click here for file
