# Supplementary material for: Self-assembly of copper and cobalt complexes with hierarchical size and catalytic properties for hydroxylation of phenol
Source: Nanoscale Res Lett. 2011 Aug 8;6(1):484. doi: 10.1186/1556-276X-6-484 (PMC3211998; doi:10.1186/1556-276X-6-484)
Supplement: Additional file 2 — Studies of Various Reaction Parameters. supp1.doc, 56 K. [file 1556-276X-6-484-S2.DOC]

Supporting Information for:

Self-assembly of Copper and Cobalt Complexes with Hierarchical Size and Catalytic Properties for Hydroxylation of Phenol

Huaze Dong ^[a]^, Wenbo Tao ^[b]^, Jianhong Bi *^,[a]^, Victoria Milway ^[c]^, Zhiqiang Xu ^[d]^, Shengyi Zhang ^[b]^, Xiangchun Meng ^[b]^, Wentao Bi ^[b]^, Jian Li ^[a]^ and Meng Li^[b]^

*^a^ Department of Chemistry and Chemical Engineering, Hefei Normal University, Hefei 230061, P.R.China；*

*^b^ School of Chemistry and Chemical Engineering, Anhui University, Hefei 230039, P.R.China*

*^c^ School of Chemistry, University of Glasgow, University Ave., GlasgowG12 8QQ, United Kingdom*

*^d^ SKC Power Tech, Inc., 850 Clark Drive, Mt. Olive, NJ 07828, U.S.A.*

**Catalysis Experiments:**

1.1 Catalytic activity

The hydroxylation of phenol in aqueous medium was carried out using three catalysts Mcps-1(nanorods, microrods and macrorods). For each run, phenol (0.2 g) and 30% H_2_O_2_ (1.0 mL ) were mixed in 40 ml of distilled water. To this solution, 10mg of a Mcps-1 catalyst was added. The reaction mixture was heated for 5 h at 50 ^◦^C with continuous stirring in a water bath. The progress of the reactions was monitored by liquid chromatography [1]. The catechol and hydroquinone were observed along with phenol in all cases. It was found that the three catalysts give 73.0%, 45.8% and 36.2% conversion of phenol respectively, under similar conditions. All three catalysts are more selective for hydroquinone formation. To determine suitable reaction conditions for maximum conversion, studies of various reaction parameters were performed as described in the following sections. Since the Mcps-1(nanorods) catalyst gave the maximum conversion of phenol, it is used as a representative catalyst for further studies.

1.2. Effect of temperature

The performance of the catalyst Mcps-1(nanorods) was monitored at five different temperatures viz. 30, 40, 50, 60 and 70 ^◦^C in five different reaction sets. Phenol (0.2 g,), 30% H_2_O_2_ (1.0 mL) were dissolved in 40 mL of distilled water and 10mg of catalyst Mcps-1(nanorods) was added to it. The reactions were run at each temperature over a period of 5 h. The results are shown in Table 1. It is seen that at 50 ^◦^C there is 73.1% conversion of phenol. On increasing the temperature to 70 ^◦^C no significant change was observed. The turn over frequencies of the reaction at 50, 60 and 70 ^◦^C were practically the same (Table 1). At 50^◦^C temperature the HQ : CAT ratio is also highest. So 50 ^◦^C is the optimal temperature for higher phenol conversion as well as catechol formation.

| Reaction temperature(^◦^C) | Phenol conversion(%) | HQ/CAT ratio |
| --- | --- | --- |
| 30 | 25.21 | 1.46 |
| 40 | 45.47 | 2.80 |
| 50 | 73.08 | 3.81 |
| 60  70 | 71.10  71.13 | 3.70  3.65 |

1.3 Effect of amount of catalyst

Amount of catalyst has a significant effect on the percentage conversion of phenol. Phenol (0.2 g) and 30% H_2_O_2_ (1.0 mL) were dissolved in 40 ml of distilled water. Three different amounts of catalyst Mcps-1 viz. 5mg, 10mg and 20mg were used to study the effect of the amount of catalyst on percent conversion of phenol. The reaction was carried out at 50 ^◦^C in all three cases. A reaction without catalyst under similar conditions showed 1.7 % conversion of phenol. From the results as shown in Table 2, it is seen that 5mg catalyst gives 35.13% conversion of phenol and that with 10mg of catalyst the percentage conversion of phenol increased to 73.1%. Further increase in amount of catalyst to 20mg resulted in only a slight change in percentage conversion of the phenol, and so 10mg of catalyst is found to be optimal.

| Catalyst weight (mg) | Phenol conversion at 50 ^◦^C after 5h(%) | HQ/CAT ratio |
| --- | --- | --- |
| 0 | 1.7 | 1.16 |
| 5 | 35.13 | 3.21 |
| 10 | 73.08 | 3.81 |
| 20 | 75. 01 | 3.82 |

1.4 Effect of H_2_O_2_ concentration

Effect of H_2_O_2_ concentration on the oxidation of phenol was studied and the results are shown in Table 3. Four different sets of molar ratios of hydrogen peroxide to phenol viz. 0.5:1, 3:1, 6:1 and 12:1 were used, in 40 ml double distilled water with a fixed amount of 0.2 g of phenol and 10mg of catalyst Mcps-1. The reaction was carried out at 50 ^◦^C for 5 h. When the H_2_O_2_: phenol molar ratio was 12:1 and 6:1 the percentage conversion of phenol was observed to be 75.0% and 73.1%, respectively. In case of 0.5:1 molar ratio, the percentage conversion of phenol was 11.7%. The product selectivity of catechol increased with a decrease in molar ratio of H_2_O_2_: phenol. When the H_2_O_2_: phenol molar ratio was changed from 6:1 to 12:1 the increase in conversion of phenol was only about 2%. On the basis of these results it can be concluded that on balancing higher conversion of phenol and H_2_O_2_ efficiency, 6:1 molar ratio of H_2_O_2_: phenol was optimal.

| H_2_O_2_:C_6_H_5_OH molar ratio | Phenol conversion at 50 ^◦^C after 5h(%) | HQ/CAT ratio |
| --- | --- | --- |
| 0.5:1 | 11.7 | 1.16 |
| 3:1 | 35.1 | 3.21 |
| 6:1 | 73.1 | 3.81 |
| 12:1 | 75. 0 | 3.82 |

1.5 Effect of reaction time

The effect of reaction time on the catalyzed hydroxylation of phenol by H_2_O_2_ in aqueous medium was studied. Phenol (0.2 g), 30% H_2_O_2_ (1.0 mL) and MCPs-1 catalyst (10 mg) were combined in 40 mL distilled water. The reaction was carried out at 50 ^◦^C for 7 h with continuous stirring. It was found that conversion of phenol increases rapidly in the beginning of reaction and a conversion of 73.12% was achieved in 5h. No appreciable change in conversion of phenol was recorded on running the reaction for 5 h. When the reaction was allowed to continue, 75.35% conversion was observed at the end of 7h and the selectivity for catechol and hydroquinone remained unchanged.

| Time  hour | Phenol conversion at 50 ^◦^C (%) | HQ/CAT ratio |
| --- | --- | --- |
| 1 | 20.3 | 2.94 |
| 3 | 45.37 | 3.36 |
| 5 | 73.12 | 3.82 |
| 7 | 75. 35 | 3.46 |

Reference.

[1] Chromatography column: ZORBAX Eclipse XDB-C18 4.6x150 mm, mobile phase: 1% HAc-MeOH (1:1, v/v), mobile phase velocity: 0.8 ml/min, column temperature: 30 °C, sample volume: 20 ul, wavelength of ultraviolet detector: 277 nm.
